# Neuropsychiatric symptoms and dementia development: a 15-year population-based study

**DOI:** 10.1016/j.tjpad.2026.100596

**Published:** 2026-05-15

**Authors:** Francesca Remelli, Giulia Grande, Serhiy Dekhtyar, Erika J Laukka, Caterina Trevisan, Stefano Volpato, Laura Fratiglioni, Federico Triolo

**Affiliations:** a Aging Research Center, Department of Neurobiology, Care Sciences and Society, Karolinska Institutet and Stockholm University, Tomtebodavägen 18 A, 17177 Solna, Sweden; b Department of Medical Sciences, University of Ferrara, Via Savonarola 9, 44121 Ferrara, Italy; c Stockholm Gerontology Research Center, Sveavägen 155, 113 46 Stockholm, Sweden; d Orthogeriatric Unit, University Hospital of Ferrara, Via Aldo Moro 8, 44124 Ferrara, Italy

**Keywords:** Neuropsychiatric symptoms, Mild behavioral impairment, Dementia, Alzheimer’s disease, Aged

## Abstract

**Background:**

Mild Behavioral Impairment (MBI) has been proposed to detect neuropsychiatric symptoms (NPS) associated with dementia development, but evidence from population-based settings is limited.

**Objectives:**

To *(i)* investigate the association between NPS in late life and the onset of dementia over 15 years in community-dwelling older adults, and *(ii)* test the interplay of NPS and Cognitive Impairment, No Dementia (CIND) in dementia development.

**Methods:**

2597 dementia-free individuals aged 60+ from a longitudinal population-based cohort underwent cognitive assessments over 15 years. Thirty clinically-assessed NPS were mapped into five domains and, within each domain, a z-score was computed from the sum of the NPS’s points. MBI was identified when the z-score was above 1.5 standard deviations (SDs) in at least one of 5 neuropsychiatric domains. Based on a cognitive battery, CIND was defined as scoring ≥1.5 SDs below age-specific means in at least one cognitive domain. Dementia was diagnosed by DSM-IV criteria following standardized procedures.

**Results:**

MBI, present in 16.1% of the sample, was associated with a higher hazard of incident dementia over 15 years (multi-adjusted hazard ratio [HR] 1.68, 95% confidence interval [CI] 1.31–2.17). Decreased motivation and social inappropriateness were the domains associated with incident dementia (HR 2.23, 95%CI 1.59–3.14 and HR 3.29, 95%CI 1.83–5.94, respectively). Compared to those with neither, individuals with either MBI (HR 1.37, 95%CI 1.00–1.90) or CIND (HR 2.22, 95%CI 1.73–2.84) had increased dementia incidence, especially when co-occurring (HR 4.41, 95%CI 3.04–6.39).

**Conclusions:**

Late life NPS, especially with co-occurring cognitive impairment, was associated with a higher dementia incidence.

## 1 Introduction

Neuropsychiatric symptoms (NPS) are notable clinical features of dementia, highly prevalent after disease onset, and associated with patient’s suffering [1] Their presence is one of the most distressing aspects of neurocognitive disorders, significantly affecting both patients and caregivers [2,3] Further, NPS have been linked with an increased speed of cognitive and functional decline in people with dementia [4] Beyond cognitive deficits, NPS in dementia carry a high clinical burden, leading to reduced quality of life for the individual and complicating clinical management, resulting in higher healthcare costs [5,6]

Studies on NPS have also been conducted during the preclinical stages of dementia, namely when cognitive symptoms are absent or mild [2,7,8] The main hypothesis is that, similar to cognitive changes, late-onset NPS might precede dementia diagnosis, potentially being prodromal features of disease manifestation [7,9,10] In this regard, five specific neuropsychiatric domains have been proposed as particularly relevant for detecting dementia development: decreased motivation, affective dysregulation, impulse dyscontrol, social inappropriateness, and abnormal perception or thought content [11] These have been integrated in the construct of Mild Behavioral Impairment (MBI), a condition proposed to identify older individuals with newly-onset, sustained, but sometimes subtle NPS associated with a higher risk of dementia. While MBI has mostly been studied in the clinical setting (i.e., considering individuals with subjective or objective cognitive symptoms)[9,[12], [13], [14]], evidence on its association with future dementia from the community, where early detection strategies could be implemented, and over long observation periods remains limited.

Understanding the occurrence of MBI in the general population, its co-occurrence with Cognitive Impairment, No Dementia (CIND) and association with the development of dementia is critical for several reasons. First, recognizing and addressing NPS in this stage may support the timely detection and adoption of strategies to alleviate symptom burden for both affected older people and caregivers. Further, it may aid healthcare providers in improving the early diagnosis of dementia. Given the current phase of discovery and clinical implementation of disease-modifying therapies, the prodromal phase of dementia, whether characterized by CIND, MBI, or both, represents a critical window of opportunity for implementing strategies that may reduce dementia development.

Using data from a population-based study with 15 years of follow-up, this study aimed to: (i) quantify the occurrence of NPS in late life, (ii) examine the association between NPS and incident dementia, and (ii) assess the interplay of NPS and CIND in relation to dementia development.

## 2 Methods

### 2.1 Study population

This study was conducted in the Swedish National Study on Aging and Care in Kungsholmen (SNAC-K), an ongoing longitudinal population-based study of individuals aged 60 years and older living in the Kungsholmen district of Stockholm, Sweden [15] Of 5111 randomly sampled individuals from 11 age cohorts (60, 66, 72, 78, 81, 84, 87, 90, 93, 96, and 99+), 3363 (participation rate: 73.3%) were assessed at baseline (2001–2004) through a nurse interview and an extensive medical and neuropsychological examination. The younger cohorts (<78 years) were followed up every 6 years, while the older ones (≥78 years) every 3 years. The present study included data between baseline and wave 6 (2016–2019) with an observational period up to 15 years. From the 3363 baseline participants, those with dementia (n = 240), intellectual disability (n = 1), those who refused to undergo the medical examination (n = 10), and those with missing data in the neuropsychological battery (n = 515) were excluded (Figure S1), resulting in an analytical sample of 2597 participants. For the present study, we considered two additional analytical subsamples: the subgroup of cognitively intact participants (i.e., without CIND and with baseline MMSE ≥27; N = 1909), and the subgroup of those presenting CIND at baseline (N = 609).

All participants provided written informed consent, directly or through their proxy for those with cognitive impairment. The SNAC-K study was approved by the Karolinska Institutet Ethics Committee and the Regional Ethical Review Board in Stockholm in accordance with the Helsinki Declaration and its amendments.

### 2.2 Neuropsychiatric profiles

At baseline, NPS were assessed during the medical examination through a subset of the Comprehensive Psychiatric Rating Scale (CPRS), for a total of 24 symptoms [16] CPRS is an instrument to assess NPS on a 0–6 scale, with good applicability and reliability in older populations [17] Eight additional behavioral symptoms related to changes in personality in the previous two years were further investigated by the physician (see Table S1 for complete symptom list). In line with the MBI framework and previously published algorithms[13,18], the collected NPS were mapped into five neuropsychiatric domains: decreased motivation, affective dysregulation, impulse dyscontrol, social inappropriateness and abnormal perception or thought content (see Table S1 for details of the conversion matrix). For each NPS, 0 points were given for absent symptom (0–1 on the CPRS scale), 1 point for mild symptom (2–3 on the CPRS scale) and 2 points for severe symptom (4–6 on the CPRS scale), according to their duration, frequency and impact on the individual’s function [16] Regarding the personality changes, 1 point was given for each change referred. In case of missing data (Table S2), the NPS was considered absent (0 points).

Within each domain, the sum score of the symptoms was z-standardized, and the neuropsychiatric domain was considered as present with a z-score above the mean by 1.5 standard deviations (SD). The presence of a neuropsychiatric profile (i.e., Mild Behavioral Impairment [MBI]) was considered when at least one domain was affected, as suggested by ISTAART-AA criteria [11]

### 2.3 Cognitive impairment, no dementia

The presence of CIND was based on the neuropsychological battery, which incorporated Trial Making Test Part B to assess frontal abilities, word recall to evaluate episodic memory, mental rotation for visuo-spatial functions, category and letter fluency for language, and digit cancellation and pattern comparison for perceptual speed. Baseline CIND was defined as scoring ≥1.5 SDs below age-specific means in at least one cognitive domain among those not meeting the diagnostic criteria for dementia [19]

### 2.4 Dementia diagnosis

Dementia was ascertained according to DSM-IV-TR diagnostic criteria[20] in a standardized procedure at baseline and at every follow-up. Briefly, two physicians involved in the SNAC-K examination made two independent diagnoses; in case of disagreement, a neurologist, external to the data collection process, made the final diagnosis. Further, dementia diagnoses were integrated through clinical charts and the Cause of Death Register for participants without a previous dementia diagnosis in SNAC-K who died in the period between the follow-ups. Further, dementia due to Alzheimer’s Diseases (AD) was clinically diagnosed according to the National Institute of Neurological and Communicative Diseases and Stroke/Alzheimer’s Disease and Related Disorders Association criteria and explored as secondary outcome [21]

### 2.5 Covariates

During the baseline nurse interview, sociodemographic data on age, sex, education (operationalized as elementary school, high school and university) and civil status (married vs. unmarried) were collected. Lifestyle behaviors were assessed in terms of smoking (ever if current or former smoker vs. never if never smoker) and alcohol use (no/occasional if once a month or less frequently, light/mild if 2–4 times a month and heavy consumption if 2 times a week or more). Psychiatric history (presence vs. absence) was recorded during the baseline medical examination based on lifetime history of depression, anxiety, phobia, sleep disorders or psychosis. In terms of somatic chronic conditions, the following diseases were assessed through the medical examination, medication review, and medical health records within the Swedish National Patient Register, and considered in the analyses: [22] chronic heart disease (i.e., chronic heart failure, ischemic heart disease, or atrial fibrillation), cerebrovascular disease, diabetes, chronic obstructive pulmonary disease, chronic kidney disease and solid neoplasms. A Body mass index (BMI) value ≤18.5 Kg/m2 was considered a proxy for undernutrition. Lastly, data on current pharmacological therapy were collected during the medical interview and operationalized as the total number of drugs and the use of psychoactive medications (antidepressants [ATC N06A], anxiolytics [ATC N05B], antipsychotics [ATC N05A] and lithium [ATC N05AN]).

### 2.6 Statistical analysis

Continuous variables were presented using mean and standard deviation (SD), while frequencies and percentages were reported for discrete variables. Participants’ characteristics at baseline were compared according to the presence of MBI through the Chi-Square or Fisher tests, depending on the type of variable.

The association between MBI (and its individual components) and incident dementia was assessed through Cox regression models. The Incidence Rates (IR) per 1000 person-years were calculated across exposure groups by dividing the number of incident dementia cases by the total person-time at risk. Participants were considered at risk until their dementia diagnosis, death, or end of follow-up, whichever came first. The strength of the associations was expressed as Hazard Ratios (HRs) and 95% Confidence Intervals (95%CIs). The proportional hazard assumption was assessed both graphically and through the goodness-of-fit test. Two models with increasing number of covariates were tested: Model 1, adjusted for age, sex, education, and psychiatric history; and Model 2, further adjusted for marital status, smoking, alcohol use, body mass index, chronic heart disease, cerebrovascular disease, and chronic obstructive pulmonary disease. Last, the interplay between MBI and cognitive impairment in the development of dementia was explored by incorporating baseline CIND and MBI in an indicator variable, resulting in four profiles (*No MBI-No CIND; Isolated MB; Isolated CIND; MBI+CIND*).

*Sensitivity analyses.* To test the effect of the length of observation period on the association between MBI and dementia, analyses were repeated considering (i) a shorter follow-up (6 years), and (ii) the whole follow-up (15 years) excluding participants developing dementia within the first 6 years (n = 172). To ensure that the association between MBI and dementia was not fully explained by the inclusion of participants with chronic psychiatric disorders, we conducted an additional analysis excluding participants with previous psychiatric history (n = 514). To examine the role of symptom severity, the main analyses were additionally repeated distinguishing, within the no-MBI group, participants with neuropsychiatric symptoms not meeting MBI criteria (NPS/MBI−) from those with no neuropsychiatric symptoms at all (No NPS), resulting in three exposure categories: No NPS, NPS/MBI−, and MBI (NPS/MBI+).

All analyses were conducted performed using Software R (version 4.2.2), and a p-value <0.05 was considered statistically significant.

## 3 Results

The analytical sample included 2597 free-dementia participants with a mean age of 72.2 (SD 9.9) and 61.4% were women. Compared to the study population, those excluded due to missing data at baseline were older, more frequently women, less educated, with lower cognitive levels, and higher use of chronic psychoactive drugs (Table S3).

**Fig. 1:**
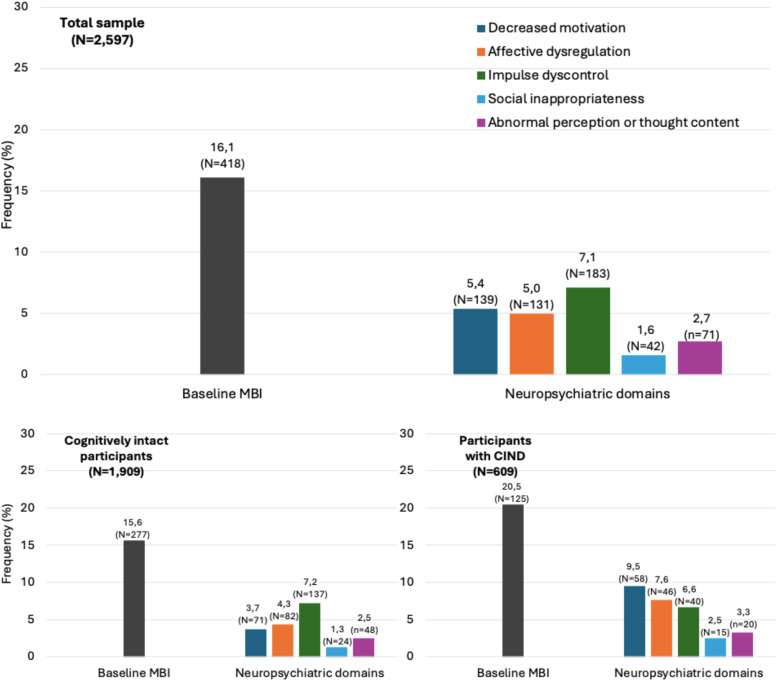
Prevalence of baseline MBI and of the five neuropsychiatric domains in the overall sample, in cognitively intact participants, and in participants with CIND. MBI: Mild Behavioral Impairment; CIND: Cognitive Impairment No Dementia.

At baseline, 16.1% (N = 418) of the participants had MBI; the prevalence of the single domains is reported in Fig. 1.

Table 1 presents the baseline characteristics based on MBI status. Compared to those without, participants with MBI were more likely to be older, unmarried and current smokers, while no differences were observed for sex and educational attainment. Moreover, those with MBI had a higher number of chronic diseases; particularly, chronic heart disease, cerebrovascular disease, diabetes, COPD and chronic kidney disease. Further, they were more likely to have a previous psychiatric history and use chronic psychoactive medications compared to those without MBI.

**Table 1:** Participants’ characteristics according to baseline MBI status.

	No MBI N = 2179	MBI N = 418	p
Age (mean ± SD)	72.0 (9.9)	73.1 (10.1)	0.032
Women	1344 (61.7)	250 (59.8)	0.506
Education			
*Elementary school*	301 (13.8)	58 (13.9)	0.769
*High school*	1062 (48.7)	211 (50.5)	
*University*	816 (37.4)	149 (35.6)	
Civil status (married)	1066 (49.0)	178 (42.7)	0.021
Smoking (ever)	306 (14.1)	78 (18.7)	0.021
Alcohol consumption			
*no/occasional*	638 (29.4)	149 (35.8)	0.033
*light/mild*	1160 (53.4)	202 (48.6)	
*heavy*	374 (17.2)	65 (15.6)	
BMI, kg/m^2^ (mean ± SD)	25.9 (4.1)	25.7 (4.2)	0.400
MMSE score (mean ± SD)	28.9 (1.5)	28.6 (1.9)	<0.001
No. of medications (mean ± SD)	3.5 (3.1)	4.8 (3.6)	<0.001
Use of psychoactive drugs, yes	218 (10.0)	96 (23.0)	<0.001
*Antidepressants*	130 (6.0)	62 (14.8)	<0.001
*Anxiolytics*	91 (4.2)	47 (11.2)	<0.001
*Antipsychotics*	18 (0.8)	11 (2.6)	0.003
*Lithium*	6 (0.3)	5 (1.2)	0.025
Chronic diseases			
*Chronic heart diseases*^a^	445 (20.4)	121 (28.9)	<0.001
*Cerebrovascular diseases*	113 (5.2)	42 (10.0)	<0.001
*Diabetes*	176 (8.1)	48 (11.5)	0.029
*COPD*	86 (3.9)	34 (8.1)	<0.001
*Chronic kidney disease*	651 (29.9)	153 (36.6)	0.008
*Solid neoplasms*	194 (8.9)	34 (8.1)	0.678
No. of somatic chronic diseases (mean ± SD)	3.5 (2.2)	4.0 (2.3)	<0.001
Previous psychiatric history, yes	379 (17.4)	135 (32.3)	<0.001
Baseline CIND, yes	484 (22.2)	125 (29.9)	0.001

If not specified, tables report number (%).

MBI: Mild Behavioral Impairment; BMI: Body Mass Index, MMSE: Mini Mental State Examination; COPD: Chronic Obstructive Pulmonary Disease; CIND: Cognitive Impairment No Dementia.

a Chronic heart diseases, defined as ischemic heart disease, heart failure or atrial fibrillation.

Table 2, Table 3 report the associations between MBI and its specific domains with dementia incidence, respectively. During a mean follow-up of 8.0 years (SD 5.4; Figure S2 for dropouts over the follow-up), 405 (15.6%) of participants developed dementia. In multi-adjusted models, participants with MBI had an increased hazard of dementia (HR 1.68, 95%CI 1.31–2.17) compared to those without MBI. The presence of this association was confirmed in both subsamples of cognitively intact participants (n = 1909; HR 1.53, 95%CI 1.07–2.19) and of those with CIND (n = 609; HR 1.87, 95%CI 1.25–2.80). When exploring dementia due to AD as secondary outcome, the association with MBI was confirmed, although attenuated in the cognitively intact subgroup (Table S4). Regarding the specific neuropsychiatric domains, decreased motivation and social inappropriateness were independently associated with a higher likelihood of dementia (HR 2.23, 95%CI 1.59–3.14 and HR 3.29, 95%CI 1.83–5.94, respectively) in the fully adjusted model.

**Table 2:** Adjusted Hazard ratios (HR) Association between baseline MBI status and 15-year dementia.

		Model 1 Basic adjusted HR (95%CI)	Model 2 Fully adjusted HR (95%CI)
	*IR per 1000 p-y*	*N/Cases* *2597/405*	*N/Cases* *2520/387*
No MBI	17.37	REF	REF
MBI	30.11	**1.78 (1.40–2.28)**	**1.68 (1.31–2.17)**
Cognitively intact participants, N = 1,909^a^		*1909/227*	*1878/221*
No MBI	12.75	REF	REF
MBI	19.09	**1.55 (1.10–2.20)**	**1.53 (1.07–2.19)**
***Participants with CIND, N=609***		*609/148*	*567/137*
No MBI	31.88	REF	REF
MBI	61.44	**2.17 (1.49–3.17)**	**1.87 (1.25–2.80)**

MBI: Mild Behavioral Impairment; HR: Hazard Ratio; 95%CI: 95% Confidence Interval; IR per 1000 p-y: absolute incidence rates per 1000 person-years.

Bold formatting indicates p < 0.05.

a Intact participants were those without baseline CIND and with MMSE ≥27 Model 1: adjusted for age, sex, education and psychiatric history; Model 2: Model 1 + marital status, smoking, alcohol, body mass index, chronic heart diseases, cerebrovascular diseases and chronic obstructive pulmonary disorders.

**Table 3:** Association between individual neuropsychiatric domains and 15-year dementia.

		Model 1 Basic adjusted HR (95%CI)		Model 2 Fully adjusted HR (95%CI)	
	No. of Events	Separate models	Mutually adjusted model	Separate models	Mutually adjusted model
*N/Cases*		*2597/405*	*2597/405*	*2520/387*	*2520/387*
Decreased motivation	48	**2.83** **(2.09–3.84)**	**2.63** **(1.90–3.63)**	**2.35** **(1.70–3.24)**	**2.23** **(1.59–3.14)**
Affective dysregulation	32	**1.61** **(1.11–2.34)**	1.30 (0.87–1.95)	**1.59** **(1.10–2.34)**	1.43 (0.94–2.17)
Impulse dyscontrol	26	1.19 (0.79–1.78)	0.82 (0.53–1.26)	1.11 (0.74–1.65)	0.85 (0.55–1.31)
Social inappropriateness	15	**3.97** **(2.35–6.71)**	**3.62** **(2.13–6.17)**	**3.25** **(1.81–5.82)**	**3.29** **(1.83–5.94)**
Abnormal perception	10	1.59 (0.85–2.99)	1.57 (0.83–2.96)	1.35 (0.67–2.74)	1.40 (0.68–2.85)

MBI: Mild Behavioral Impairment; HR: Hazard Ratio; 95%CI: 95% Confidence Interval.

Bold formatting indicates p < 0.05.

Model 1: adjusted for age, sex, education and psychiatric history; Model 2: + marital status, smoking, alcohol, body mass index, chronic heart diseases, cerebrovascular diseases and chronic obstructive pulmonary disorders.

**Fig. 2:**
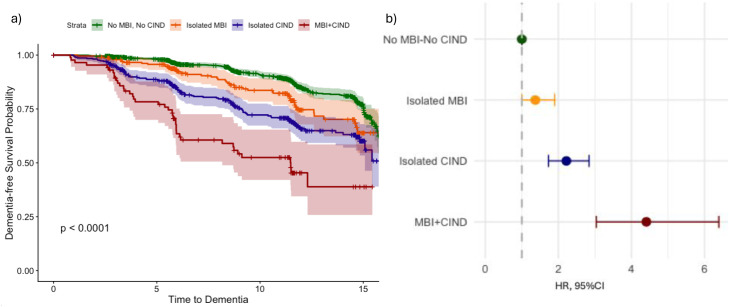
A) Kaplan-Meier curve of dementia development over 15 years according to the MBI, CIND, and their co-presence (a); B) Forest plot on the association between the MBI, CIND, and their co-presence with incident dementia from a Cox regression model (reference group: no MBI-no CIND). MBI: Mild Behavioral Impairment; CIND: Cognitive Impairment No Dementia; HR: Hazard Ratio; 95%CI: 95% Confidence Interval. The model showed in Figure B) was adjusted for age, sex, education, psychiatric history, marital status, smoking, alcohol, body mass index, chronic heart diseases, cerebrovascular diseases and chronic obstructive pulmonary disorders.

Fig. 2 shows the association between the MBI and CIND, and their co-presence, with the development of dementia (see Table S5 for participants’ characteristics at baseline based on MBI/CIND status). Compared to those with neither MBI nor CIND, participants with isolated MBI showed a 37% increased hazard of dementia (HR 1.37, 95%CI 1.00–1.90), those with isolated CIND reported over a two-fold higher hazard of dementia (HR 2.22, 95%CI 1.73–2.84) and participants with both MBI and CIND had more than four-fold higher hazard of dementia (HR 4.41, 95%CI 3.04–6.39). Further, the combined profile *MBI+CIND* was associated with an increased hazard of dementia (HR 2.09, 95%CI 1.41–3.09) when the *Isolated CIND* profile was set as the reference group (data not shown).

### 3.1 Sensitivity analyses

The pattern of results was consistent when we considered a shorter follow-up (6 years; Table S6, column A). After the exclusion of individuals who developed dementia within the first 6 years of follow-up (N = 172), the association between MBI and 15-year dementia was further confirmed in the overall sample, but was attenuated in both subgroups of cognitively intact participants or those with CIND (Table S6, column B). After excluding participants with psychiatric history (N = 514), the pattern of results was preserved (Table S7). When the no-MBI group was further distinguished into participants with neuropsychiatric symptoms (NPS/MBI−) and those with no symptoms at all (No NPS), only MBI was independently associated with incident dementia across the overall sample and both subgroups, whereas NPS/MBI− showed a non-significant gradient toward increased risk (Table S8).

## 4 Discussion

In this longitudinal population-based study of Swedish older adults, NPS, classified through the MBI framework, were observed in nearly one-fifth of participants and showed to be associated with a higher incidence of dementia over a 15-year follow-up. This association was evident in both cognitively intact individuals and those with CIND at baseline. Among the specific neuropsychiatric domains, decreased motivation and social inappropriateness were independently associated with an increased dementia risk. Furthermore, participants with co-occurring MBI and CIND had a faster progression to dementia over time, compared to those with neither condition as well as those with isolated CIND. Overall, these findings suggest that older individuals presenting with late-onset NPS experience an accelerated cognitive decline, underscoring the importance of investigating new NPS as a potential early marker of dementia onset.

In our sample, the prevalence MBI was 16%, which was lower than reported in other population-based cohorts [23,24] However, estimates of MBI prevalence remain inconsistent across studies, ranging from 5.8% to 85.3%[25], likely due to differences in neuropsychiatric assessment tools and MBI operationalizations. While most of the studies have used the Neuropsychiatric Inventory (NPI) [26] or its shorter form (Neuropsychiatric Inventory Questionnaire, NPI-Q)[13,27], these instruments may be less sensitive in assessing NPS in individuals without dementia compared to scales such as the MBI Checklist (MBI-C), a tool specifically created to screen for MBI [18] Nevertheless, the MBI-C cutoffs to identify MBI are still under debate, adding further variability in the reported prevalences [[28], [29], [30], [31]] Identifying a sensitive MBI-C cutoff suggestive of a behavioral prodrome of dementia is critical, considering the high frequency of NPS in older population [32,33] Further, differences in study populations, particularly the inclusion of individuals with varying degree of cognitive impairment, might influence NPS prevalence, as up to 45% of those with mild cognitive impairment have been reported to present MBI [14] Given the potential prognostic relevance of MBI and its impact on caregivers[13,34], precise quantification of NPS in population-based cohorts remains a key priority.

Our results confirmed that the presence of late-life NPS is associated with the development of dementia among community-dwelling older adults. Overall, this finding aligns with evidence from previous population-based studies, where the presence of NPS, measured with NPI, was associated with the development of mild cognitive impairment in individuals with normal cognition [35] Further, in another population-based study using the same instrument to assess NPS, people with both NPS and mild cognitive impairment presented with increased conversion rates to dementia[2], a finding also reported in another memory-clinic-based study [36] Yet, as previously mentioned, the use of different methods to assess NPS has limited the comparability and generalizability of results across studies [2,7,8] To overcome this, the construct of MBI was developed in the attempt to standardize the identification of NPS in late life linked with a higher risk of dementia [14,34] Standardizing the assessment of NPS through MBI may improve early detection strategies and facilitate research in this area. While diverse late life NPS have been linked with increased speed of cognitive and functional decline in people before dementia onset[1,2,5], they could be considered as early manifestation of neurodegenerative disorders [37] This hypothesis may also be supported by our findings, as MBI was associated with dementia diagnosed within 6 years of follow-up, but not with cases diagnosed between 6 and 15 years, suggesting that MBI may represent a prodromal sign of dementia. Moreover, consistent with the association between MBI and incident AD observed in our study, growing evidence, although mostly from memory-clinic samples, reports that people with MBI present higher levels of AD biomarkers, especially phosphorylated-tau 181, which also tends to increase over time [[38], [39], [40], [41]] In addition, few studies on brain imaging reported specific neurodegenerative markers (e.g. atrophy of the entorhinal cortex and hippocampus) among individuals presenting with MBI [42] All these findings support MBI as an early sign of dementia, similar to CIND, but from a neuropsychiatric perspective [37] Given the long preclinical phase of dementia, future studies on neurobiological correlates of MBI in population-based settings are needed to clarify the role of this construct in relation to the cognitive continuum and the underlying neuropathology.

In our study, the two individual neuropsychiatric domains associated with dementia development were those more related to a frontal lobe syndrome, namely decreased motivation and social inappropriateness [43] Apathy has been long associated with an increased risk of dementia, especially AD, and has been linked to dysfunctions in fronto-striatal circuitry in neurodegenerative disorders [44] A previous study using the MBI construct confirmed the association between decreased motivation and dementia[45,46], while other studies have identified impulse dyscontrol as the domain most associated with conversion to dementia [47] This discrepancy might be due to the clinical settings in which most of the studies have been carried out, leading to the inclusion of participants with different patterns in NPS reflecting prodromal phases of specific dementia subtypes. Notably, impulse dyscontrol and disinhibition are typical early symptoms of frontotemporal dementia[48], but they are also frequently detected in the behavioral AD variant[49], suggesting that differences in the underlying neuropathological profiles of participants may influence the heterogeneous findings observed across studies.

Based on our findings, the presence of MBI was associated with a higher progression to dementia in people with CIND, and the co-presence of both conditions was linked to the fastest rate of dementia development. This is in line with previous studies showing that specific NPS in people with CIND were associated with steeper cognitive decline, increased dementia risk, and lower likelihood of reversion to normal cognition [14,19,50,51] A recent clinic-based study further confirmed these findings, reporting that participants with both mild cognitive impairment and MBI had a lower likelihood of reversion to normal cognition compared to those without [19] Moreover, similarly with isolated CIND, we found that participants with isolated MBI had an increased hazard of future dementia, even if with a weaker strength when compared to isolated CIND or co-occurring MBI and CIND. Overall, these findings support the role of the routine assessment of NPS to identify people with an increased risk of developing dementia, as specific neuropsychiatric patterns may signal accelerated disease progression [34] Incorporating neuropsychiatric evaluations alongside cognitive assessments in clinical settings could enhance prognostication, particularly for individuals already experiencing cognitive impairment [14] Further, although NPS are more prevalent in individuals with co-occurring cognitive impairment, their assessment in cognitively intact older adults may still be informative for identifying subgroups at elevated dementia risk, rather than as a broad population-wide screening approach. This supports the need for greater public and primary care awareness to facilitate timely diagnostic investigations and follow-up. As disease-modifying therapies for dementia advance toward clinical implementation, expanding the concept of at-risk conditions for dementia to include non-cognitive symptoms such as NPS is essential for targeting earlier phases of the disease and optimizing care strategies.

This study has several strengths. First, it relies on a large population-based cohort of older adults. Second, it features an extended follow-up period of up to 15 years. Third, it includes extensive cognitive and psychopathological assessments conducted by trained physicians and neuropsychologists using standardized protocols over the study period. Fourth, dementia was ascertained according to diagnostic criteria in a standardized procedure involving three independent physicians.

However, some limitations need to be considered. First, as in other studies[9,10,13], MBI was diagnosed retrospectively by adapting the psychopathological symptoms available in SNAC-K to the MBI domains proposed in the literature. Most studies retrospectively derived MBI using the Neuropsychiatric Inventory-Questionnaire (NPI-Q)[27], a clinical tool created to screen NPS in people with dementia [13] Consequently, NPI-Q might be less effective at capturing symptoms in the general population, as well as under- or over-estimate the prevalence of NPS due to the limited range of symptoms it covers. In contrast, our study integrated an extensive range of clinically assessed psychiatric symptoms and personality changes[16], which maximizes the capture of relevant MBI domains. Even so, specific domains such as abnormal perception may be less represented, given the population-based setting and the relatively healthy profile of participants. Second, similar to most available studies, our neuropsychiatric assessment did not meet the onset timing of NPS indicated by ISTAART criteria [11] However, some of the symptoms included in the MBI diagnosis, namely those reflecting recent behavioral changes, may have increased the likelihood of identifying new and persistent manifestations. In addition, to verify that our findings were not driven by previous psychiatric disorders, analyses were repeated after excluding participants with long-term psychiatric history, and the pattern of results remained consistent. Third, NPS were assessed at baseline, consistent with the conceptualization of MBI as a risk marker for dementia onset. Future work should examine how changes in NPS over time relate to dementia risk. Fourth, although we used DSM-IV-TR criteria[20], mild dementia cases may not have been captured. Fifth, the relatively long-time assessments maybe have led to imprecision in the timing of dementia onset, especially among the younger cohorts, and precluded a more granular examination of how the association between NPS and dementia evolves across shorter follow-up windows. Lastly, the exclusion of participants with missing neuropsychological data, the relatively healthy profile of the cohort, and differential attrition over follow-up may have underrepresented the frailest individuals, limiting generalizability and potentially underestimating the observed associations. In conclusion, our population-based study showed an association between MBI and incident dementia in both cognitively intact individuals and those with CIND. Among specific neuropsychiatric domains, decreased motivation and social inappropriateness emerged as significant predictors of dementia. Finally, participants with coexistent MBI and CIND showed the faster rate of converting to dementia compared to those with neither. Overall, these findings strengthen the need for routinely assessing NPS in older adults as they may provide crucial information on dementia risk, particularly when they co-occur with cognitive impairment.

## Funding

The Swedish Ministry of Health and Social Affairs, the participating County Councils and Municipalities, and the Swedish Research Council (ongoing/current grant: 2021-00178) financially supported the Swedish National Study on Aging and Care in Kungsholmen (SNAC-K). F.T. acknowledges Demensfonden, Lindhes Advokabyrå (LA2024-0033), and the Swedish Research Council for Health, Working Life and Welfare (FORTE, 2025-01519) for supporting this work.

## Consent statement

All participants provided informed consent.

## Declaration of generative AI and AI-assisted technologies in the writing process

The Authors have used AI-assisted tools for language editing only. No AI tools were used for data analysis, interpretation, or scientific content generation.

## CRediT authorship contribution statement

**Francesca Remelli:** Writing – original draft, Visualization, Methodology, Formal analysis, Data curation, Conceptualization. **Giulia Grande:** Writing – original draft, Supervision, Methodology, Conceptualization. **Serhiy Dekhtyar:** Writing – review & editing, Supervision, Conceptualization. **Erika J Laukka:** Writing – review & editing, Methodology, Conceptualization. **Caterina Trevisan:** Writing – review & editing, Methodology, Conceptualization. **Stefano Volpato:** Writing – review & editing, Supervision, Conceptualization. **Laura Fratiglioni:** Writing – review & editing, Methodology, Conceptualization. **Federico Triolo:** Writing – review & editing, Visualization, Supervision, Methodology, Formal analysis, Data curation, Conceptualization.

## Declaration of competing interest

The authors declare that they have no known competing financial interests or personal relationships that could have appeared to influence the work reported in this paper.

## Appendix Supplementary materials

Image, application 1
