# Supplementary material for: Neuropsychiatric symptoms and dementia development: a 15-year population-based study
Source: J Prev Alzheimers Dis. 2026 May 15;13(7):100596. doi: 10.1016/j.tjpad.2026.100596 (PMC13200101; doi:10.1016/j.tjpad.2026.100596)
Supplement: Supplementary file 1 [file mmc1.docx]

**Supplementary material**

**
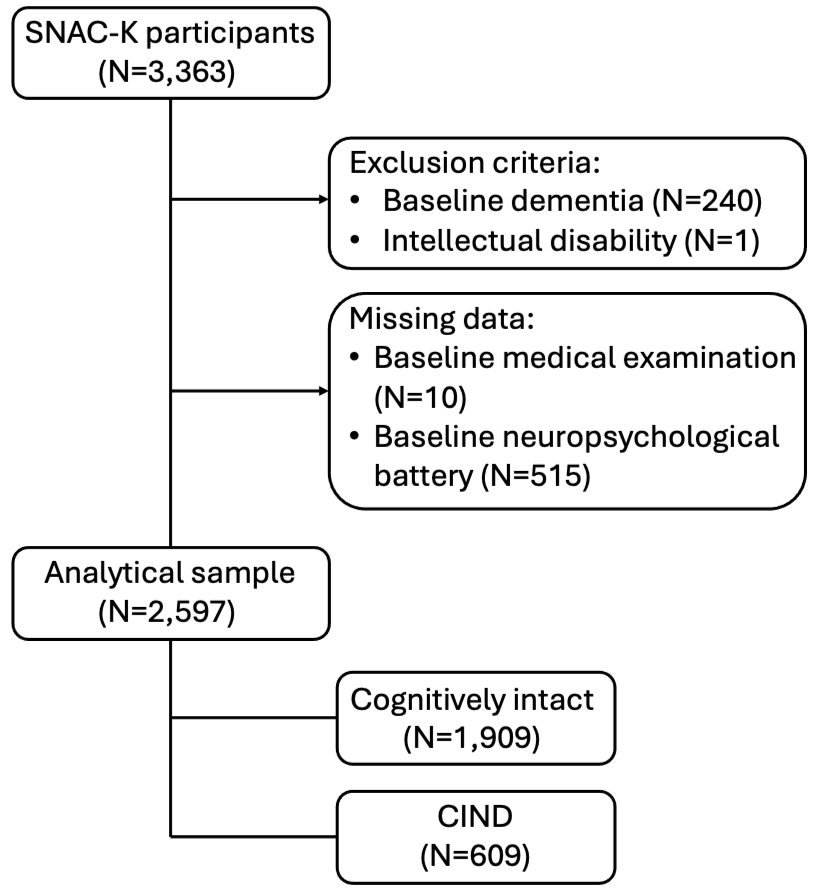
**

**Figure S1.** Flowchart of the study participants.

*CIND : Cognitive Impairment, No Dementia*


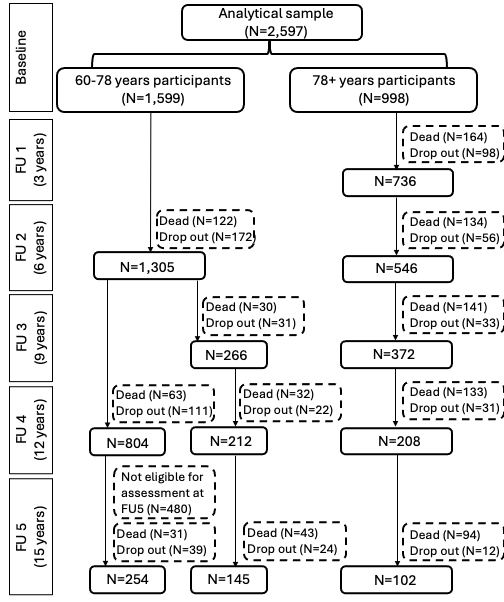


**Figure S2.** Flowchart of the study participation over 15 years.

**Table S1.** Neuropsychiatric symptoms in SNAC-K and related MBI domains.

| **MBI domains** | **NPS items in SNAC-K** | **Assessment tools** | **Original score** |
| --- | --- | --- | --- |
| **Decreased motivation** | Social Seclusion | Reported CPRS | 0-6 |
|  | Indecision |  | 0-6 |
|  | Lassitude |  | 0-6 |
|  | Reduced Speech | Observed CPRS | 0-6 |
|  | Slowness of Movement |  | 0-6 |
|  | More or less talkative? | Changes in Personality | 0-1 |
|  | More or less reclusive? |  | 0-1 |
|  | More or less listless? |  | 0-1 |
|  | More difficulty taking initiative than before? |  | 0-1 |
|  | More trouble making decisions than before? |  | 0-1 |
| **Affective dysregulation** | Inner Tension | Reported CPRS | 0-6 |
|  | Inability to feel |  | 0-6 |
|  | Hypochondriasis |  | 0-6 |
|  | Worrying over trifles |  | 0-6 |
|  | Sense of Worthlessness |  | 0-6 |
|  | Pessimistic Thoughts |  | 0-6 |
|  | Suicidal Thoughts |  | 0-6 |
|  | Reduced sexual interest |  | 0-6 |
|  | Sadness or Apparent Sadness* | Reported CPRS and Observed CPRS | 0-6 |
|  | More or less worried? | Changes in Personality | 0-1 |
| **Impulse dyscontrol** | Hostile Feelings | Reported CPRS | 0-6 |
|  | Autonomic Disturbances |  | 0-6 |
|  | Agitation | Observed CPRS | 0-6 |
|  | More or less cross? | Changes in Personality | 0-1 |
|  | More or less agitated? |  | 0-1 |
| **Social inappropriateness** | Difficulty gauging social boundaries | Observed CPRS | 0-2 |
|  | Disinhibition |  | 0-2 |
| **Abnormal perception or thought content** | Morbid Jealousy | Reported CPRS | 0-6 |
|  | Fabrication | Observed CPRS | 0-2 |
|  | Suspicion |  | 0-6 |

*MBI: Mild Behavioral Impairment; NPS: neuropsychiatric symptoms; SNAC-K: Swedish National Study on Aging and Care in Kungsholmen; CPRS: Comprehensive Psychiatric Rating Scale*

******Sadness was considered present if recorded in either reported or observed CPRS, using the highest score available for severity*

**Table S2.** Prevalence of missing data for neuropsychiatric symptoms.

|  | **Whole sample**  **(N=2,597)** |
| --- | --- |
| **1) Comprehensive Psychiatric Rating Scale (CPRS)** |  |
| ***Reported CPRS*** |  |
| Sadness | 7 (0.3) |
| Inability to feel | 19 (0.7) |
| Lassitude | 25 (1.0) |
| Pessimism | 15 (0.6) |
| Inner tension | 9 (0.3) |
| Suicidal thoughts | 16 (0.6) |
| Hostile feelings | 16 (0.6) |
| Hypochondria | 17 (0.7) |
| Worrying over trifles | 13 (0.5) |
| Sense of worthless | 20 (0.8) |
| Social seclusion | 15 (0.6) |
| Indecision | 21 (0.8) |
| Reduced sexual interest | 700 (27.0) |
| Autonomic disturbances | 37 (1.4) |
| Morbid jealousy | 851 (32.8) |
| ***Observed CPRS*** |  |
| Apparent sadness | 16 (0.6) |
| Reduced speech | 12 (0.5) |
| Slowness of movement | 12 (0.5) |
| Agitation | 19 (0.7) |
| Suspicion | 12 (0.5) |
| Difficulty gauging social boundaries | 11 (0.4) |
| Disinibition | 11 (0.4) |
| Fabrication | 11 (0.4) |
| **2) Changes in personality** |  |
| More or less listless | 33 (1.3) |
| More difficulty taking initiative | 29 (1.1) |
| More or less worried | 29 (1.1) |
| More or less reclusive | 31 (1.2) |
| More trouble making decisions | 32 (1.2) |
| More or less cross | 29 (1.1) |
| More or less talkative | 29 (1.1) |
| More or less agitated | 43 (1.7) |

*If not specified, tables report number (%)*

*CIND: Cognitive Impairment No Dementia; CPRS: Comprehensive Psychiatric Rating Scale*

**Table S3.** Comparison of baseline characteristics between included and excluded SNAC-K participants based on missing data.

|  | **Included**  **N=2,597** | **Excluded**  **N=525** | **p** |
| --- | --- | --- | --- |
| Age (mean ± SD) | 72.2 (9.9) | 80.6 (11.6) | <0.001 |
| Women | 1594 (61.4) | 377 (73.2) | <0.001 |
| Education |  |  |  |
| *Elementary school* | 359 (13.8) | 132 (26.3) | <0.001 |
| *High school* | 1273 (49.0) | 271 (54.1) |  |
| *University* | 965 (37.2) | 98 (19.6) |  |
| Civil status (married) | 1244 (48.0) | 145 (28.5) | <0.001 |
| Smoke habit | 384 (14.9) | 68 (13.8) | 0.573 |
| Alcohol consumption |  |  |  |
| *No/occasional* | 787 (30.4) | 282 (57.4) | <0.001 |
| *Light/mild* | 1362 (52.6) | 149 (30.3) |  |
| *Heavy* | 439 (17.0) | 60 (12.2) |  |
| BMI, kg/m^2^ (mean ± SD) | 25.8 (4.1) | 24.4 (4.3) | <0.001 |
| MMSE (mean ± SD) | 28.8 (1.5) | 26.7 (3.9) | <0.001 |
| No. of medications (mean ± SD) | 3.7 (3.2) | 4.7 (3.7) | <0.001 |
| Use of psychoactive drugs | 314 (12.1) | 124 (24.2) | <0.001 |
| Chronic diseases |  |  |  |
| *Chronic heart diseases^a^* | 541 (20.8) | 187 (36.3) | <0.001 |
| *Cerebrovascular diseases* | 155 (6.0) | 59 (11.5) | <0.001 |
| *Diabetes* | 224 (8.6) | 51 (9.9) | 0.396 |
| *COPD* | 120 (4.6) | 33 (6.4) | 0.109 |
| *Chronic kidney disease* | 804 (31.0) | 232 (45.0) | <0.001 |
| *Solid neoplasms* | 228 (8.8) | 51 (9.9) | 0.465 |
| No. of somatic chronic diseases (mean ± SD) | 3.57 (2.22) | 4.72 (2.69) | <0.001 |
| Previous psychiatric history | 514 (19.8) | 112 (21.7) | 0.342 |
| Baseline CIND | 609 (23.5) | - | - |

*If not specified, tables report number (%)*

*MBI: Mild Behavioral Impairment; BMI: Body Mass Index, MMSE: Mini Mental State Examination; COPD: Chronic Obstructive Pulmonary Disease; CIND: Cognitive Impairment No Dementia*

*^a^Chronic heart diseases*, *defined as ischemic heart disease, heart failure and atrial fibrillation*

**Table S4.** Association between baseline MBI status and 15-year dementia due to Alzheimer’s Disease.

| ***Total sample, N=2,597*** | **Fully adjusted Model**  **HR (95%CI)** |
| --- | --- |
| *N/Cases*  MBI | *2,520/204*  **1.81 (1.27-2.58)** |
| ***Cognitively intact participants, N=1,909****^a^* | **Fully adjusted Model**  **HR (95%CI)** |
| *N/Cases*  MBI | *1,878/114*  1.54 (0.90-2.61) |
| ***Participants with CIND, N=609*** | **Fully adjusted Model**  **HR (95%CI)** |
| *N/Cases*  MBI | *567/75*  **2.11 (1.23-3.62)** |

*MBI: Mild Behavioral Impairment; CIND: Cognitive Impairment; No Dementia; AD: Alzheimer’s Disease; HR: Hazard Ratio; 95%CI : 95% Confidence Interval*

*Bold formatting indicates p<0.05*

*^a^Intact participants were those without baseline CIND and MMSE >27.*

*Models adjusted for age, sex, education, psychiatric history, marital status, smoking, alcohol, body mass index, chronic heart diseases, cerebrovascular diseases and chronic obstructive pulmonary disorders.*

**Table S5.** Participants’ characteristics by four profiles of MBI and CIND.

|  | **No MBI,**  **No CIND**  **N=1,695,**  **65.3%** | **Isolated**  **MBI**  **N=293, 11.3%** | **Isolated**  **CIND**  **N=484,**  **18.6%** | **MBI+**  **CIND**  **N=125, 4.8%** | **p** |
| --- | --- | --- | --- | --- | --- |
| Age (mean ± SD) | 71.4 (9.8) | 72.8 (9.9) | 74.3 (9.7) | 73.9 (10.6) | <0.001 |
| Women | 1,020 (60.2) | 169 (57.7) | 324 (66.9) | 81 (64.8) | 0.022 |
| Education |  |  |  |  |  |
| *Elementary* | 183 (10.8) | 31 (10.6) | 118 (24.4) | 27 (21.6) | <0.001 |
| *High school* | 807 (47.6) | 140 (47.8) | 255 (52.7) | 71 (56.8) |  |
| *University* | 705 (41.6) | 122 (41.6) | 111 (22.9) | 27 (21.6) |  |
| Civil status (married) | 874 (51.6) | 139 (47.6) | 192 (39.8) | 39 (31.2) | <0.001 |
| Smoking | 228 (13.5) | 46 (15.7) | 78 (16.3) | 32 (25.6) | 0.002 |
| Alcohol consumption |  |  |  |  |  |
| *No/occasional* | 183 (10.8) | 31 (10.6) | 118 (24.4) | 27 (21.6) | <0.001 |
| *Light/mild* | 807 (47.6) | 140 (47.8) | 255 (52.7) | 71 (56.8) |  |
| *Heavy* | 705 (41.6) | 122 (41.6) | 111 (22.9) | 27 (21.6) |  |
| BMI, kg/m^2^ (mean ± SD) | 25.9 (4.0) | 25.8 (3.9) | 25.7 (4.2) | 25.4 (4.8) | 0.605 |
| MMSE (mean ± SD) | 29.1 (1.1) | 29.0 (1.2) | 28.1 (2.0) | 27.5 (2.6) | <0.001 |
| No. of medications (mean ± SD) | 3.4 (3.1) | 4.8 (3.7) | 3.9 (3.3) | 4.7 (3.4) | <0.001 |
| Use of psychoactive drugs | 160 (9.4) | 66 (22.5) | 58 (12.0) | 30 (24.0) | <0.001 |
| *Antidepressants* | 93 (5.5) | 48 (16.4) | 37 (7.7) | 14 (11.2) | <0.001 |
| *Anxiolytics* | 71 (4.2) | 31 (10.6) | 20 (4.1) | 16 (12.8) | <0.001 |
| *Antipsychotics* | 13 (0.8) | 6 (2.0) | 5 (1.0) | 5 ( 4.0) | 0.003 |
| *Lithium* | 4 (0.2) | 2 (0.7) | 2 (0.4) | 3 (2.4) | 0.004 |
|  |  |  |  |  |  |
| Chronic diseases |  |  |  |  |  |
| *Chronic heart diseases^a^* | 300 (17.7) | 78 (26.6) | 124 (25.6) | 39 (31.2) | <0.001 |
| *Cerebrovascular disease* | 80 (4.7) | 22 (7.5) | 33 (6.8) | 20 (16.0) | <0.001 |
| *Diabetes* | 116 (6.8) | 27 (9.2) | 60 (12.4) | 21 (16.8) | <0.001 |
| *COPD* | 63 (3.7) | 25 (8.5) | 23 (4.8) | 9 (7.2) | 0.002 |
| *Chronic kidney disease* | 497 (29.3) | 105 (35.8) | 154 (31.8) | 48 (38.4) | 0.032 |
| *Solid neoplasms* | 147 (8.7) | 27 (9.2) | 47 (9.7) | 7 (5.6) | 0.533 |
| No. of somatic chronic diseases (mean ± SD) | 3.4 (2.1) | 3.9 (2.1) | 3.8 (2.3) | 4.4 (2.6) | <0.001 |
| Previous psychiatric history | 298 (17.6) | 92 (31.4) | 81 (16.7) | 43 (34.4) | <0.001 |

*If not specified, tables report number (%)*

*MBI: Mild Behavioral Impairment; BMI: Body Mass Index, MMSE: Mini Mental State Examination; COPD: Chronic Obstructive Pulmonary Disease; CIND: Cognitive Impairment No Dementia*

*^a^Chronic heart diseases*, defined *as ischemic heart disease, heart failure and atrial fibrillation*

**Table S6.** Association between baseline MBI and dementia development over 6- and 15-year follow-up.

| ***Total sample*** | **Fully adjusted Model**  **HR (95%CI)** | |
| --- | --- | --- |
|  | **A** | **B** |
|  | **6-year follow-up**  **(N=2,597)** | **15-year follow-up excluding dementia cases within the first 6 years**  **(N=2,425)** |
| *N/Cases*  MBI | *2,520/156*  **2.01 (1.40-2.88)** | *2,364/231*  1.14 (0.99-2.03) |
| ***Cognitively intact participants^a^*** | **Fully adjusted Model**  **HR (95%CI)** | |
|  | **6-year**  **(N=1,909)** | **15-year**  **(N=1,852)** |
| *N/Cases*  MBI | *1,878/114*  **1.87 (1.00-3.51)** | *1,827/170*  1.36 (0.88-2.10) |
| ***Participants with CIND*** | **Fully adjusted Model**  **HR (95%CI)** | |
|  | **6-year**  **(*N=609*)** | **15-year**  **(N=514)** |
| *N/Cases*  MBI | *567/75*  **2.26 (1.41-3.66)** | *482/52*  1.28 (0.58-2.79) |

*MBI: Mild Behavioral Impairment; HR: Hazard Ratio; 95%CI: 95% Confidence Interval*

*Bold formatting indicates p<0.05*

*^a^Intact participants were those without baseline CIND and MMSE >27*

*Models adjusted for age, sex, education, psychiatric history, marital status, smoking, alcohol, body mass index, chronic heart diseases, cerebrovascular diseases and chronic obstructive pulmonary disorders*

**Table S7.** Association between baseline MBI status and 15-year dementia, excluding participants with previous psychiatric history.

| ***Total sample, N=2,083*** | **Fully adjusted Model**  **HR (95%CI)** |
| --- | --- |
| *N/Cases* | *2,021/324* |
| MBI | **1.69 (1.26-2.26)** |
| ***Cognitively intact participants, N=1,535^a^*** | **Fully adjusted Model**  **HR (95%CI)** |
| *N/Cases* | *1,513/185* |
| MBI | 1.46 (0.96-2.23) |
| ***Participants with CIND, N=485*** | **Fully adjusted Model**  **HR (95%CI)** |
| *N/Cases* | *449/115* |
| MBI | **2.06 (1.31-3.23)** |

*MBI: Mild Behavioral Impairment; HR: Hazard Ratio; 95%CI: 95% Confidence Interval*

*Bold formatting indicates p<0.05*

*^a^Intact participants were those without baseline CIND and MMSE >27*

*Model adjusted for age, sex, education, psychiatric history, marital status, smoke, alcohol, body mass index, chronic heart diseases, cerebrovascular diseases and chronic obstructive pulmonary disorders*

**Table S8.** Association between subthreshold neuropsychiatric symptoms (NPS/MBI−) and Mild Behavioral Impairment (NPS/MBI+) and 15-year incident dementia, compared to individuals with no neuropsychiatric symptoms (No NPS), in the overall sample and cognitive subgroups.

|  | **Fully adjusted model** | | |
| --- | --- | --- | --- |
|  | **HR** | **95% Lower CI** | **95% Upper CI** |
| ***Total sample, N=2,597*** |  |  |  |
| *No NPS, N=594* | REF | - | - |
| *NPS/MBI-, N=1,585* | 1.25 | 0.96 | 1.64 |
| *NPS/MBI+, N=418* | **1.99** | **1.44** | **2.75** |
|  |  |  |  |
| ***Cognitively intact participants, N=1,909*** |  |  |  |
| *No NPS, N=450* | REF | - | - |
| *NPS/MBI-, N=1,182* | 1.18 | 0.83 | 1.67 |
| *NPS/MBI+, N=277* | **1.74** | **1.11** | **2.71** |
|  |  |  |  |
| ***Participants with CIND, N=609*** |  |  |  |
| *No NPS, N=124* | Ref | Ref | Ref |
| *NPS/MBI-, N=360* | 1.28 | 0.79 | 2.09 |
| *NPS/MBI+, N=125* | **2.26** | **1.30** | **3.91** |

*MBI: Mild Behavioral Impairment; HR: Hazard Ratio; 95%CI: 95% Confidence Interval*

*Bold formatting indicates p<0.05*

*^a^Intact participants were those without baseline CIND and MMSE >27*

*Model adjusted for age, sex, education, psychiatric history, marital status, smoke, alcohol, body mass index, chronic heart diseases, cerebrovascular diseases and chronic obstructive pulmonary disorders*
